# Complete Genome Sequence of Bifidobacterium longum subsp. *longum* JCM7052

**DOI:** 10.1128/MRA.01193-20

**Published:** 2021-04-08

**Authors:** Isamu Yamamoto, Yumiko Ueno, Miho Geshi, Yoshishige Inagaki, Toshitaka Odamaki, Kiyotaka Fujita

**Affiliations:** a Faculty of Home Economics, Kobe Women’s University, Kobe, Japan; b Faculty of Literature, Kobe Women’s University, Kobe, Japan; c Next Generation Science Institute, Morinaga Milk Industry Co. Ltd., Zama, Kanagawa, Japan; d Faculty of Agriculture, Kagoshima University, Kagoshima, Japan; University of Maryland School of Medicine

## Abstract

We report the complete genome sequence of Bifidobacterium longum subsp. *longum* JCM7052, isolated from human feces in Japan. This strain has the capability of growing on and utilizing gum arabic as an energy source. The complete genome is 2,273,627 bp long, with 1,929 protein-coding genes and 59.9 mol% G+C content.

## ANNOUNCEMENT

Bifidobacteria are Gram-positive, non-spore-forming, multimorphogenic rod-shaped, and strictly anaerobic bacteria. Some species of the genus *Bifidobacterium* are used as probiotics, which are defined as live microorganisms that, when administered in adequate amounts, confer a health benefit on the host (1). Among these species, Bifidobacterium longum subsp. *longum* is a stable and dominant population in the human gastrointestinal tract, and the genes in its genome vary depending on the habitat, such as the types and quantity of carbohydrates ingested by the host (2). In the genus *Bifidobacterium*, some strains of B. longum subsp. *longum* and B. adolescentis have been shown to ferment gum arabic as the carbohydrate source (3). We observed that B. longum subsp. *longum* JCM7052, which was isolated from the feces of an adult human by Mitsuoka (4), can grow on gum arabic (5).

B. longum subsp. *longum* JCM7052 was grown under anaerobic conditions in *Bifidobacterium* medium (5), and DNA was extracted using the cetyltrimethylammonium bromide (CTAB) method (6). A sequence library was prepared with the TruSeq DNA sample prep kit (Illumina; FC-121-1001) as described in the manufacturer’s instructions. Sequencing was conducted using a next-generation sequencer, the Illumina HiSeq 2000 (San Diego, CA), and generated 100-bp paired-end reads. A total of 116,426,984 reads and 11,759 Mb of sequence data (∼2,500× coverage) was obtained after a quality check of the sequence reads, performed using CASAVA version 1.8.1, a software provided by Illumina. A total of 36 contigs were assembled using Velvet version 1.2.10 (7), and GapCloser version 1.10 was used to close the gaps that emerged during the scaffolding process, conducted using SOAPdenovo (8). The contigs were placed in order compared with the genome sequence of B. longum subsp. *longum* JCM1217 (GenBank accession number AP010888) using Mauve (9, 10). Since each contig contained a part of the IS*21* insertion sequence (IS) family or rRNA operon in its terminal regions, the primers for PCR were designated referring to the sequences adjacent to the IS and rRNA operon (Table 1), and PCR amplification was performed (Bio-Rad T100 thermal cycler). The amplicons obtained were subjected to sequencing by means of primer walking on an Applied Biosystems 3730 DNA sequencer using a BigDye Terminator version 3.1 cycle sequencing kit (Applied Biosystems, Foster City, CA). The amplicon sequences were assembled into ordered contigs by visual examination. Finally, a circular sequence was obtained. This sequence was annotated using the DDBJ Fast Annotation and Submission Tool (DFAST) (11), and some products were identified using a BLAST (12) search of DDBJ service analytical tools.

**TABLE 1:** PCR primer pairs used for gap closing between contigs*^a^*

Target contig	Forward primer	Reverse primer
NODE4-10	5′-GCGAGACGATAGTCGAGCCATAAGG-3′	5′-CCTATCGGTCGATGATATGACCGCC-3′
NODE10-6	5′-CCTCGCTATAGTGCTCGCCCTGCTC-3′	5′-GTTTCATCGACGCCGACCCGCTCAT-3′
NODE6-7	5′-GGTGTCGTGTCTGGCCACCAATACG-3′	5′-TCTGCGGAATTCAGCGCGACATGCC-3′
NODE7-5	5′-CAGATGGACAGCGCTGGCAGTCACA-3′	5′-GGAGAAGTTCATGCCGGACAGCAGC-3′
NODE5-3	5′-TCCAAGTTCGACGACATGGAGCCGG-3′	5′-AAAGCCTGGGATTGAGCCCGTTGGC-3′
NODE3-1	5′-GAGACGCGAAAGGACTCATGGTGGC-3′	5′-ACAGGAGGTTCGCGTTCTTGTCCGG-3′
NODE1-9	5′-GCCTACAGAGCGGGTCATTACGAGC-3′	5′-AGGTACGGGTACGAACGCTCCAACG-3′
NODE9-8	5′-GGGCCGACAAGCTGAAAACTCTCGC-3′	5′-AAGCCAGTTTCTGGCTCCCCTCTGG-3′
NODE8-2	5′-GGTCGCGTTGTCCATCACGATCACG-3′	5′-CATGCAGGTCGATTTCGGCATGGCC-3′
NODE2-4	5′-GGAGGCCAACATCGTGTCCATCGAC-3′	5′-CGTGACGCCATCGACGGATTGGAGA-3′
NODE6-11	5′-CCAAGAGGTTTCTGGCTCCCCTCTT-3′	5′-GGGCGAAGCCTGTCGTTTCAACGAT-3′
NODE9-2	5′-TCGATGATGAGAAGTCGGGCCTTGC-3′	5′-CATGCAGGTCGATTTCGGCATGGCC-3′

a PCR was done under the following conditions: 95°C for 5 min followed by 30 cycles at 94°C for 30 sec, 45°C to 55°C for 30 sec, and 72°C for 3 to 6 min.

The complete genome of B. longum subsp. *longum* JCM7052 is 2,273,627 bp long and has a G+C content of 59.9 mol%. The genome contains 1,929 putative proteins and carries 4 rRNA operons, 56 tRNAs, and 1 transfer-messenger (tmRNA). Eight gene clusters found in the genome of strain JCM7052 were not found in B. longum subsp. *longum* JCM1217, which is a strain that cannot assimilate gum arabic. These genes may provide useful insights into the physiological properties of strain JCM7052.

### Data availability.

This whole-genome project has been deposited in DDBJ/ENA/GenBank under accession number AP022379. The BioProject accession number is PRJDB9197. The raw sequence reads have been deposited in the DRA under accession number DRA011367.
